# The Emerging Role of Liquid Biopsy in Gastric Cancer

**DOI:** 10.3390/jcm10102108

**Published:** 2021-05-13

**Authors:** Csongor György Lengyel, Sadaqat Hussain, Dario Trapani, Khalid El Bairi, Sara Cecilia Altuna, Andreas Seeber, Andrew Odhiambo, Baker Shalal Habeeb, Fahmi Seid

**Affiliations:** 1 Head and Neck Surgery, National Institute of Oncology Hungary, 1122 Budapest, Hungary; 2 North West Cancer Center, Altnagelvin Hospital, Londonderry BT47 6SB, UK; oncologysh@gmail.com; 3 European Institute of Oncology, IRCCS, 20141 Milan, Italy; dario.trapani@ieo.it; 4 Cancer Biomarkers Working Group, Oujda 60000, Morocco; k.elbairi@ump.ac.ma; 5 Oncomédica C.A., Medical Oncology Department, Caracas 1010, Venezuela; altunamujica.md@gmail.com; 6 Department of Hematology and Oncology, Comprehensive Cancer Center Innsbruck, Medical University of Innsbruck, 6020 Innsbruck, Austria; andreas.seeber@tirol-kliniken.at; 7 Unit of Medical Oncology, Department of Clinical Medicine, University of Nairobi, Nairobi 30197, Kenya; andrewodhiambo@gmail.com; 8 Department of Medical Oncology, Shaqlawa Teaching Hospital, Shaqlawa, Erbil 44005, Iraq; bakershalal@gmail.com; 9 School of Medicine and Health Sciences, Hawassa University, Hawassa 1560, Ethiopia; fahmizeek@gmail.com

**Keywords:** liquid biopsy, circulating tumor cell, cfDNA, ctDNA, metastatic gastric cancer, epithelial–mesenchymal transition, resistance to treatment, HER2-inhibition, VEGFR-inhibition, immunotherapy, response monitoring

## Abstract

(1) Background: Liquid biopsy (LB) is a novel diagnostic method with the potential of revolutionizing the prevention, diagnosis, and treatment of several solid tumors. The present paper aims to summarize the current knowledge and explore future possibilities of LB in the management of metastatic gastric cancer. (2) Methods: This narrative review examined the most recent literature on the use of LB-based techniques in metastatic gastric cancer and the current LB-related clinical trial landscape. (3) Results: In gastric cancer, the detection of circulating cancer cells (CTCs) has been recognized to have a prognostic role in all the disease stages. In the setting of localized disease, cell-free DNA (cfDNA) and circulating tumor DNA (ctDNA) qualitative and quantitative detection have the potential to inform on the risk of cancer recurrence and metastatic dissemination. In addition, gastric cancer-released exosomes may play an essential part in metastasis formation. In the metastatic setting, the levels of cfDNA show a positive correlation with tumor burden. There is evidence that circulating tumor microemboli (CTM) in the blood of metastatic patients is an independent prognostic factor for shorter overall survival. Gastric cancer-derived exosomal microRNAs or clonal mutations and copy number variations detectable in ctDNA may contribute resistance to chemotherapy or targeted therapies, respectively. There is conflicting and limited data on CTC-based PD-L1 verification and cfDNA-based Epstein–Barr virus detection to predict or monitor immunotherapy responses. (4) Conclusions: Although preliminary studies analyzing LBs in patients with advanced gastric cancer appear promising, more research is required to obtain better insights into the molecular mechanisms underlying resistance to systemic therapies. Moreover, validation and standardization of LB methods are crucial before introducing them in clinical practice. The feasibility of repeatable, minimally invasive sampling opens up the possibility of selecting or dynamically changing therapies based on prognostic risk or predictive biomarkers, such as resistance markers. Research is warranted to exploit a possible transforming area of cancer care.

## 1. Introduction

The term liquid biopsy simultaneously encompasses a group of significantly different techniques, emerging as a tool that clinical practitioners can use to diagnose cancer, assess prognosis, identify targetable alterations, predict the effectiveness of treatments, and monitor tumor burden and therapeutic resistance. Among solid tumors, the potential for liquid biopsy has been most thoroughly studied in colorectal cancer, non-small cell lung cancer, and breast cancer and less explored in gastric cancer [1,2,3]. In our introduction, following a brief description of gastric cancer’s prognostic and predictive biomarkers, we provide a brief overview of the history and applications of liquid biopsy. It is not the purpose of this article to describe the technical and procedural background and limitations of liquid biopsy, which would go beyond the scope of this review. Readers may also refer to some of the excellent recent reviews on this topic [1,2,3].

### 1.1. Overview of the Prognostic and Predictive Tissue Biomarkers in Gastric Cancer

Stomach cancer is the fifth most prevalent tumor globally, with a worldwide incidence in increasing trend of growth [4]. According to the Global Cancer Observatory of the World Health Organization, from the 1.09 million cases diagnosed in 2020, the incidence will rise to 1.77 million by 2040 [5].

Histologically, around 90% of gastroesophageal junction cancer (GEJC) and gastric cancer cases are adenocarcinomas [6,7]. Gastric adenocarcinomas can be further divided into four categories based on The Cancer Genome Atlas (TCGA) Research Network’s molecular classification. The classification differentiated (i) chromosomally unstable, (ii) Epstein–Barr virus-induced, (iii) genomically stable, and (iv) microsatellite unstable groups [8].

Several biomarkers have prognostic or predictive value in gastric cancer, and their prevalence may vary depending on the disease stage. Both Programmed cell death ligand 1 (PD-L1) and Human epidermal growth factor receptor 2 (HER2)-positive tumors have been linked to aggressive disease and reduced survival, although some conflicting evidence still exists [9,10,11,12]. A decreased risk of lymph node metastasis, tumor invasion, and mortality has been correlated with microsatellite instability (MSI) in stomach cancer. However, the connection with improved prognosis remains unclear in light of conflicting evidence [13]. Several publications have questioned the role of neoadjuvant chemotherapy in microsatellite-instable tumors and supported a surgery-only approach. The predictive value of microsatellite instability in the clinical decision of providing perioperative chemotherapy may be prospectively confirmed by subgroup analyses of the FLOT-4 and JACCRO GC-07 studies [14,15,16,17]. In EBV-positive gastric cancers, subsequent studies have also demonstrated that PD-L1 expression is increased and associated with a decrease in survival [18,19].

The clinical benefit of immunotherapy has been reported in two subgroups of gastric cancer, in tumors with elevated PD-L1 expression and in tumors with microsatellite instability [20,21,22]. Correlation between HER2 positive status and clinical response to trastuzumab has also been observed [11]. Therefore, according to the National Comprehensive Cancer Network (NCCN)’s newest guidelines in the USA, MSI testing is suggested in all newly diagnosed patients, and HER2 and PD-L1 testing are recommended in all metastatic cases [23]. High tumor mutational burden and positive EBV status are emerging predictive biomarkers for gastric cancer immunotherapy [24,25,26].

### 1.2. A Brief Introduction to Liquid Biopsy

In 1948, Mandel and Métais published the results of discovering extracellular nucleic acids (DNA and RNA) in the blood [27]. In 1966, Leon et al. measured circulating free DNA (cfDNA) levels in 173 oncology patients and 55 healthy controls by using radioimmunoassay. The authors observed that 50% of cancer patients had normal cfDNA levels. However, they reported substantially higher levels of cfDNA in the serum of metastatic patients. In some patients, dynamic changes in cfDNA have been observed after radiation therapy by using serial sampling [28]. Nearly thirty years later, evidence has been provided that tumor cells release their DNA into the bloodstream. By parallel testing of the primary tumor and the plasma, characteristic mutations of selected genes like RAS or gene alterations in the genes involved in DNA mismatch repair (MMR) (i.e., present in the primary tumors) could also be detected in the matching plasma. This phenomenon was reported across the indications by several groups [29,30,31]. In 2008, Diehl et al. demonstrated that it is possible to molecularly detect tumor relapse in colorectal cancer by tracking individually selected tumor-specific mutations in the plasma. The authors summed this approach’s benefits in its high specificity, and the downside was to create a unique mutation-specific probe marker for each subject [32]. Simultaneously, it was recognized that tumor DNA could be isolated from many other body fluids, such as cerebrospinal fluid, saliva, pleural fluid, urine, and fecal samples [33,34,35,36]. Nowadays, liquid biopsy is composed of different biological sources such as circulating tumor microemboli (CTM), circulating tumor cells (CTC), cell-free nucleic acids, exosomes, or tumor-educated platelets (TEP) [37,38]. Additional information can be obtained by molecular characterization in addition to detecting these specific circulating factors’ presence or concentration.

The aim of this narrative review, based on this context, is to study the current landscape and the future application of liquid biopsy in metastatic gastric cancer. Therefore, we present an outline of its potential role in the diagnosis of metastatic disease and monitoring of clonal dynamics during systemic therapies together with possible future applications.

## 2. The Role of Liquid Biopsy in Gastric Cancer in the Early Detection of Metastatic Disease

Circulating free DNA (cfDNA) in the blood is mainly derived from apoptotic cells, mostly from leukocytes [39]. Invasive primary tumors, circulating tumor cells, and metastatic sites may also be the source of circulating free DNA passively or actively releasing tumor DNA into the circulation [40]. In a case-control study, enrolling 30 gastric cancer patients and 34 healthy individuals, Kim et al. demonstrated that mean plasma cfDNA levels were the lowest among healthy subjects and highest in patients with advanced gastric cancer [41]. In a larger trial, among the subset of 18 gastric cancer patients, an association has been observed between the higher level of postoperative cfDNA and recurrence [42]. One study followed the dynamics of total circulating cell-free DNA (cfDNA) levels in 73 gastric cancer patients in the postoperative period. The authors reported that plasma cfDNA levels might increase for three months after surgery, then decrease [43]. Circulating tumor DNA (ctDNA) is usually only a small portion of circulating free DNA and has recently emerged to predict recurrence or relapse in several cancer types. For example, in breast cancer, serial ctDNA sampling is reliable in identifying tumor progression or distant metastatic disease. Circulating tumor DNA may be detected from the plasma up to 1 year before radiologically detectable disease progression [44]. Other similar studies have found that liquid biopsies can also be used for surveillance of other gastrointestinal malignancies. In addition to detecting relapsed or recurrent disease, the presence of ctDNA may influence the choice of adjuvant chemotherapy [45,46].

In gastric cancer patients undergoing gastrectomy, a Japanese study has monitored somatic mutations of the TP53 (tumor protein P53) gene in plasma that were initially present in (matched) tumor samples and ctDNA (detected in 3/42 patients). Elevated TP53-mutant ctDNA levels were associated with a higher risk of disease progression; however, the number of patients that the authors could track (*n* = 3) was relatively small [47]. The hypothesis of detecting molecular residual disease (MRD) by ctDNA has been prospectively investigated in 46 patients with stage I–III gastric cancer resected with curative purpose. Baseline ctDNA was positive in 45 percent of the plasma samples and independently associated with the primary tumor’s extent (*p* = 0.006). Patients with early tumors with no gastric *muscularis propria* infiltration had no detectable pre-operative ctDNA (*p* = 0.024), irrespective of the involvement of the regional lymph nodes. Postoperative ctDNA detection was associated with disease recurrence in all patients in this study. The ctDNA positivity preceded radiological recurrence by a median of 6 months, and its positivity at any time postoperatively associated with worse disease-free and overall survival [48]. Another study analyzed the personalized cancer-specific rearrangements of 25 gastric cancer patients in the postoperative period for one year. While no correlation was found between ctDNA positivity preoperatively and cancer recurrence, the detection of ctDNA postoperatively was significantly associated with cancer recurrence in the first year (*p* = 0.029), and the median time observed between ctDNA detection and radiologic cancer recurrence was 4.05 months [49]. The Japanese MONSTAR-SCREEN study performed a serial ctDNA assay involving 540 patients with advanced solid tumors, of whom 133 had gastrointestinal (GI) tumors (48 patients had gastric tumors). Published results showed that ctDNA levels in GI tumors were significantly higher compared to non-GI tumors. One-third of the alterations detected in tumors were detected only in ctDNA, not in the tissue sample [50]. The SCRUM-Japan GOZILA (UMIN000016343) study examined the role of ctDNA-based comprehensive genomic profiling in facilitating the recruitment of patients for clinical trials compared to tissue-based detection. The use of liquid biopsy, according to the authors, reduced the lead time of testing to its one-third (11 vs. 33 days) and doubled the rate of patient enrolment into studies (9.5 vs. 4.1%). When detectable target alterations in tumors were determined using ct-DNA, the efficacy of the treatment was not inferior to that associated with tissue-based determination [51].

In gastric cancer, the detection of circulating tumor cells (CTCs) in peripheral blood may have clinical utility in monitoring tumor recurrence and metastatic spread [52]. According to a meta-analysis encompassing 2566 patients from 26 trials, the detection of CTCs was correlated with a substantial inferior effect on the patients’ overall survival in all stages [53].

In a prospective trial that has enrolled 93 patients with resectable gastric cancer, patients with CTCs ≥ 5/7.5 mL detected in postoperative blood samples had significantly inferior disease-free survival (DFS) and overall survival (OS) than those with a smaller number of CTCs. The increased number of CTCs after treatment correlated with early recurrence as well [54]. In addition to the detection and concentration of CTCs, additional methods can be used to characterize CTCs, including chromosomal abnormalities, cell surface markers, and receptors. One example of this is the CD44 positivity of the CTCs, which may have a negative prognostic significance in epithelial tumors. A prospective trial that enrolled 228 patients with resectable gastric cancer found that during the long-term follow-up, among the 99 cytokeratin-positive tumors, distant metastases were observed in half of the CD44-positive patients, compared with 19% of patients in the CD44-negative group [55]. CTCs often contain more than two copies of chromosome 8. Based on this feature, they can be identified by specific methods (e.g., SET-iFISH). According to Li et al., monitoring the therapeutic response in metastatic gastric cancer by tracking chromosome eight aneuploidy may be a useful tool [56,57]. Cancerous stem cell features, tumor cell invasiveness, and chemoresistance properties are all gained during the epithelial–mesenchymal transformation (EMT). The emergence of mesenchymal markers and a decline in epithelial markers accompany this transformation. As a result, epithelial markers are unable to identify CTCs undergoing EMT (EpCAM-based enrichment methods) [58,59,60].

The development of peritoneal metastases is relatively common, affecting one in two patients with gastric cancer. Predisposing factors for the development of peritoneal metastases are less well known. Tumor implantation, hematogenous spread, or exosomes have all been described as possible mechanisms for developing peritoneal metastasis [61]. Several authors have questioned the practical utility of using CTC identification for the early detection of peritoneal metastases. According to a prospective study involving 136 patients with advanced gastric cancer, the presence of peritoneal metastases did not correlate with the number of CTCs [57]. In contrast to CTCs, gastric cancer-released exosomes may play an essential part during the process of peritoneal metastatic spread in the transformation of the premetastatic microenvironment. Exosomes may weaken the mesothelial barrier by inducing apoptosis of the peritoneal cells and fibrosis [62]. The content of exosomes consists of proteins, lipids, and RNA (miRNA and mRNA) that are characteristic of the original cell secreting them, thus allowing further segmentation [63]. The composition of microRNA (miRNA) contained in exosomes may induce proliferation and has been reported to determine the site of metastasis formation (organotropism) [64,65]. In a bioinformatics study of exosomal miRNAs, three candidates were suggested as biomarkers for gastric cancer metastasis, namely miR-10b-5p for nodal metastasis, miR-101-3p for ovarian metastasis, and miR-143-5p for liver metastasis [66]. Another study described gastric cancer exosomes enriched with miR-106a being able to induce the formation of peritoneal metastases [67]. Among serosa-involved gastric cancer patients, reduced exosomal miR 29 levels in peritoneal lavage fluid or ascites are correlated with the enhanced risk of developing peritoneal metastases and worse OS [68]. In addition to exosomal information transfer from tumor cells, there is also crosstalk between healthy tissues and tumor cells. A proteomic study confirmed the role of omental exosomes in the growth of gastric cancer and the development of peritoneal metastases [69]. In the circulation, microRNAs can not only be found in exosomes or microvesicles, but also in a protein-bound state. MicroRNAs primarily bind to Argonaute2 (Ago2) protein or to High-density lipoprotein (HDL). As described in colon cancer, monitoring of Ago2 as well as Ago2-miRNA levels in the blood of gastric cancer patients may be a possible marker of tumor progression and response to chemotherapy [70,71,72].

## 3. The Role of Liquid Biopsy in Disease Monitoring

Circulating tumor microemboli (CTM) are clusters of CTCs in the blood, which are not only entirely composed of tumor cells. Non-cancerous cell types found in CTMs are white blood cells, fibroblasts, endothelial cells, pericytes, and platelets [73]. Several authors have described that tumor cells in CTM have specific phenotypic and molecular characteristics. In contrast to CTCs, no apoptotic signals are observed in CTMs, suggesting that the CTM clusters are not developing within the blood, but tumor cells are cleaved together from their site of origin, retaining their cell–cell connections [74]. In a study that enrolled 41 patients with treatment-naive metastatic gastric cancer, Zheng et al. examined the prognostic significance of CTM in peripheral blood samples. In a multivariate analysis, detectable CTM in the blood was an independent prognostic factor for shorter overall survival [58].

In the next section, we detail the role of liquid biopsy in the therapy follow-up and treatment resistance.

### 3.1. Liquid Biopsy in Response Monitoring or Detection of Resistance Mechanisms to Chemotherapy or Targeted Therapy

In recent years, monitoring the response to anti-cancer treatment has undergone a rapid change. Liquid biopsy has proven to be helpful in different solid malignancies for detecting new resistance mechanisms to chemotherapy and targeted therapy. However, data available for molecular mechanisms of resistance to gastric cancer treatment in this clinical setting is still scarce. Liquid biopsies may also help diagnose resistance to treatment before radiological progression and are gaining more relevance as a tool for optimizing patient care. The serum exosome proteome of metastatic gastric cancer patients was defined recently in detail by Ding et al. [75]. A study showed that, in gastric cell lines, exosomal Ribosomal Protein S3 (RPS3) secreted by cisplatin-resistant tumor cells could be taken up by cisplatin-sensitive cells and thus become chemoresistant (through activation of the PI3K-Akt-cofilin-1 signaling pathway) [76]. Another gastric cell-line experiment has identified the microRNA-501-5p (miR-501) as an inductor of doxorubicin resistance [77]. Overexpressed long noncoding RNAs (lncRNAs) may regulate chemotherapy resistance indirectly through a variety of mechanisms [78].

Two main targeted therapy groups are approved for their use in metastatic gastric cancer, namely anti-angiogenics and HER2 inhibitors. The retrospective biomarker analysis of the phase III REGARD trial examined the role of VEGFR2 (vascular endothelial growth factor receptor 2) expression on survival and the response to ramucirumab. This analysis ruled out any predictive value of the VEGFR2 expression levels and suggested a non-significant prognostic trend of shorter PFS among the high expressors. The analysis proved no relationship between the baseline concentration of serum circulating VEGF-C, VEGF-D, VEGFR1, VEGFR3 proteins, and the efficacy of ramucirumab treatment [79]. In the phase III AVAGAST trial, resistance to treatment with bevacizumab was seen among patients with lower baseline VEGF-A plasma levels and a trend towards lower survival rates. Another examined biomarker was baseline neuropilin-1 expression. Low initial neuropilin-1 expression was not only prognostic (indicating poor survival), but was predictive of the response to bevacizumab [80]. The novel VEGFR2 inhibitor apatinib appeared to antagonize the chemotherapy resistance by inhibiting the transport function of the multi-drug resistance proteins MDR1 and BCRP and could be considered in combination with platinum compounds or fluoropyrimidines. However, the clinical benefit of such strategies is still to be determined [81]. The anti-HER2 monoclonal antibody trastuzumab is a standard-of-care in treating HER-2 positive metastatic gastric cancer. Interestingly, according to a report, the examination of the HER2 status of circulating tumor cells (CTCs) reported higher positivity in CTCs (43%) compared to primary tumors (11%) [82].

Several resistance mechanisms against HER-2 inhibition have already been described [83]. One such mechanism is downregulation of the ERBB2 at a transcriptional level, leading to lower HER2 expression and failure of HER-2 inhibition strategies [84,85]. In vitro and in vivo models with gastric cancer cell lines show that the activation of several alternative pathways is an alternative mechanism for developing resistance to HER2 inhibition. The HER4–YAP1 axis is activated after chronic exposure to trastuzumab in cell cultures and translates into a higher rate of EMT and resistance to therapy. Blocking HER4 phosphorylation lowers the activity of YAP1, a downstream transcription factor directly involved in regulating the expression of HER2 and E-Cadherin [86]. Other alternative activations in resistant gastric cancer include another downstream effector of HER2, FGF3, and RAS/PI3K, and MAPK/ERK signaling pathways, mainly through the activation of other members of the ERBB family, such as EGFR or HER3. In another experimental model, simultaneous inhibition of these three receptors potentially overcame trastuzumab resistance [87]. The activation of the PI3K pathway also upregulates the factor known as metastasis-associated in colon cancer 1 (MACC1), which promotes the Warburg effect in cancer cells, resulting in a poor prognosis [88]. A clinical trial that investigated the utility of ctDNA for the detection of biomarkers of resistance to trastuzumab therapy was conducted on 39 patients with advanced gastric cancer. The authors showed a consistent correlation of clonal mutations between tumor and peripheral blood samples, identifying 32 mutations potentially related to trastuzumab resistance, and defined another valuable biomarker to monitor response to chemotherapy, the molecular Tumor Burden Index (mTBI), as an independent prognostic factor for progression-free survival [89]. The concept of serial plasma sampling for response monitoring has been proven by Wang et al. CtDNA reliably predicted antitumor response or tumor growth in 24 trastuzumab-treated patients. By tracking HER2 copy numbers’ changes, the leading mechanism of primary or acquired resistance could be differentiated (in the case of acquired resistance, HER2 copy numbers decreased during progression, compared to baseline) [90].

### 3.2. Liquid Biopsy in Response Monitoring or Detection of Resistance Mechanisms to Immunotherapy

Immunotherapy treatment of gastric cancer has shown promising activity and recently demonstrated improved survival in selected patients with metastatic disease. In the first-line setting, the use of the anti-PD1 pembrolizumab was shown to be non-inferior to the platinum-based chemotherapy in patients with HER2-negative, PD-L1 positive tumors (i.e., Combined Positive Score [CPS] equal or higher than 1, intended as the PD-L1 positive fraction of tumor and/or immune-cell), in the KEYNOTE-062 trial [91]. Such an effect seemed driven by the subset of patients with highly immune-sensitive tumors for the presence of DNA microsatellite instability (MSI). The CheckMate 649 study has tested the combination of the anti-PD1 nivolumab and chemotherapy and demonstrated a consistent benefit in the patients with CPS > 5%, establishing a role in the first-line setting [92].

Among patients failing on first-line therapy, the use of the anti-PD1 pembrolizumab also showed to be superior to paclitaxel (CPS ≥ 1 subset) for the OS in the KEYNOTE-061 trial [25]. However, the PD-L1 expression in gastric cancer has not been demonstrated to be a reproducible or univocal marker, and many concerns have been reported in confirming a predictive role of the PD-L1 CPS in selecting patients for immunotherapy [93,94]. Therefore, identifying predictive biomarkers for cancer immunotherapy is highly desirable, including less invasive diagnostic procedures and dynamic monitoring assays. The informative potential of the ctDNA in gastric tumors has been proposed for the upfront selection of patients or disease-course monitoring, either for qualitative (e.g., molecular typization) and quantitative (e.g., ctDNA change) measurements [95]. This concept is relatively new and only recently implemented in clinical research.

An emerging biomarker for tumor-agnostic utilization is the tumor mutational burden (TMB) [96]. TMB has been identified as a marker of improved survival in patients with gastric cancer receiving immune-checkpoint inhibitors [97,98].

While tissue-assessed TMB has been broadly implemented in clinical practice and research, blood-based assays (bTMB) are technically more challenging, representing an essential barrier for their validation and clinical uptake [99]. In addition, the predictive role of TMB seems partially overlapping and less significant than microsatellite instability (MSI), positivity to Epstein–Barr virus (EBV), and PD-L1 CPS [100]. These three markers are established prognostic and predictive indicators in gastric cancer [26,101,102,103]. First, the initial attempts to test circulating cancer cells for PD-L1 expression revealed challenges to differentiate them from macrophages, which might express this biomarker and be misinterpreted. Accordingly, the patient selection based on non-tissue PD-L1 assessments seems not ready for clinical utilization and requires technology improvements [104]. Exosomal PD-L1 in metastatic gastric cancer has been discovered to be an independent predictor for OS and was negatively correlated with CD4+ or CD8+ T-cell count and granzyme B levels [105,106]. Ishiba et al. examined the possibility of detecting ctRNA in the blood of 760 patients with solid tumors, including 44 cases of gastric cancer. Their study showed that it is possible to determine the mRNA of the PD-L1 gene, and quantification of PD-L1 gene expression is feasible. The authors suggest that ctRNA isolated from blood may be a potential alternative to tissue PD-L1 assay [107]. Second, applications of liquid biopsy have also been reported across several tumor types to evaluate the status of the microsatellites [108]. The ctDNA sequencing technologies have demonstrated a good concordance with the tissue-MSI assessment: 99.5% (95% CI, 98.7–99.8) and 87% (95% CI, 77–93) for low- and high-MSI, respectively [109]. Third, for the EBV status ascertainment via liquid biopsy, the evidence is less robust. Viral ctDNA can be detected in patients with EBV-positive gastric cancer by quantitative real-time polymerase chain reaction (PCR), with reasonable specificity (97%) and modest sensitivity (71%)—and mostly in patients with larger primary tumors [110]. Essentially, PCR assay detects EBV viremia in the context of EBV infection and gastric cancer, but this is not always the case for EBV-immortalized cancer cells. However, the demonstration of latent EBV in ctDNA from cancer cells is more challenging, as these cells are commonly apoptotic or necrotic, and the EBV cannot be consistently demonstrated, often not preserved and destroyed [111]. However, methylation genome markers that are recurrently present in EBV-positive tumors have been identified as surrogate indicators of EBV and may help predict the immune response [112].

Recently, the first analysis of the phase II clinical INSPIRE trial (NCT02644369; drug: pembrolizumab) has been provided. This study assesses changes between genomic and immune biomarkers with tissue- and liquid biopsy-based assays at baseline, during treatment and at progression. In the first analysis, the investigators identified tumor-specific mutations at baseline from tissue and developed a tumor-informed personalized ctDNA assay for the on-treatment monitoring [113]. The investigators confirmed a baseline prognostic significance of the ctDNA with immunotherapy, as reported in other studies, including gastric cancer cohorts [114,115]. More interestingly, the study demonstrated an informative role of the ctDNA changes to discriminate between actual disease progression and radiological pseudo-progression: when ctDNA was rising, patients were unlikely to derive a benefit from immunotherapy and, vice versa, ctDNA decline was associated with a shrinkage of the actual tumor burden [113]. This observation seems to suggest the role of liquid biopsy to confirm the disease progression and initiate immediate treatment changes in patients more unlikely to derive a clinical benefit [116]. Interestingly, ctDNA clearance was associated with sustained and durable responses to pembrolizumab, although this occurred in only a subset of patients. The radiographic response was also preceded by ctDNA clearance. Although Bratman et al. provided the first evidence for the clinical utility of ctDNA in patients receiving immunotherapy, unfortunately, it is not clear whether the study has included gastric cancer patients [113].

## 4. Ongoing Clinical Trials Using Liquid Biopsy Approaches for Gastric Cancer

Although the impact of liquid biopsy in gastric cancer is thought to be immature, significant progress is ongoing in almost all areas of gastric cancer (Table 1). The Danish CURE study is a continuous prospective cohort (NCT04576858) designed to examine the relevance of ctDNA determination in the plasma of patients with gastroesophageal cancer in different clinical cohorts: (1) Scheduled for surgical resection and perioperative chemotherapy; (2) Neoadjuvant chemoradiotherapy followed by surgical resection; (3) Definitive chemoradiotherapy with curative intent; (4) Systemic treatment to extend the patient’s life; and (5) Palliative treatment without the use of chemotherapy. A Chinese prospective cohort study (NCT04000425) uses serial sampling to evaluate the clinical use of ctDNA as a potential indicator of minimal residual disease (MRD) after radical gastrectomy. One primary endpoint of the trial is ctDNA clearance among patients with positive postoperative ctDNA; in these patient segments, the clearance of ctDNA could reflect a response to adjuvant chemotherapy. The other primary endpoint of the trial would validate the use of ctDNA for the detection of recurrent disease, measuring the time between the first ctDNA positivity and the occurrence of clinically detectable disease recurrence.

Several clinical studies from China investigate whether serial ctDNA mutation profiling may support the early diagnosis of the disease (NCT04665687) or predict recurrence in the postoperative setting (NCT02887612). Since the KEYNOTE-012 study investigated the efficacy of pembrolizumab in patients with advanced PD-L1 positive gastric cancer, a study is being performed to predict the efficacy of ctDNA for the immune-checkpoint blockade in advanced gastric cancer (NCT04053725). Three phase II trials are in progress, including one study investigating adjuvant doublet pembrolizumab and trastuzumab versus trastuzumab alone in patients with HER2+ esophagogastric cancer with persistent ctDNA following curative surgery (NCT04510285). Moreover, another phase II trial will explore the clinical value of dynamic detection of circulating tumor cells (CTCs), ctDNA, and cell-free DNA (cfDNA) in neoadjuvant chemotherapy and surgery for resectable or locally advanced gastric or gastroesophageal junction cancer settings (NCT03957564). Promisingly, GASTHER2 is another phase II trial evaluating the efficacy of adding trastuzumab to standard chemotherapy in patients with advanced HER2-negative gastric cancer and HER2-positive expression in CTCs (NCT04168931). The role of ctDNA and CTCs as a biomarker for cytoreductive surgery combined with hyperthermic intraperitoneal chemotherapy and systemic chemotherapy in gastric cancer with regional peritoneal metastasis is also being investigated in a multicenter and single-arm and phase III study (NCT03023436).

## 5. Discussion

There is a clear need for novel non-invasive diagnostic methods in both non-metastatic and metastatic gastric cancer settings, which may also capture tumor heterogeneity [117,118,119,120]. A real challenge in the early detection of gastric cancer is that its diagnosis requires an invasive procedure, and most patients are asymptomatic at an early stage [121]. Liquid biopsy may play a prominent role in early diagnosis in the future. Two studies show that elevated postoperative cfDNA may be associated with a higher risk of recurrence [42,43]. Several reports have described that ctDNA may be used as a marker of MRD and may influence the choice of adjuvant chemotherapy, and may allow personalized monitoring of progression [45,46,47]. A strong relationship between the detectability of CTCs and a substantial inferior effect on the patients’ overall survival in all stages has been reported in the literature. CTCs may also help identify high-risk patients by detecting minimal residual disease (MRD) may provide an option for risk stratifying and identifying the patients at the highest risk of recurrence [53,122]. In summary, in the nonmetastatic setting, several data show that liquid biopsy may provide valuable biomarkers to diagnose cancer early, estimate tumor volume, determine the completeness of the tumor resection and prognosis. Identification and detailed analysis of cancer-derived exosomes may be useful in identifying tumor-preferred metastatic sites, outlining the potential for a more active, organ-focused follow-up. Accurate knowledge of exosomal data transfer may open new perspectives in tumor diagnosis, monitoring of therapy, and non-invasive follow-up of the patient and may hold new therapeutic options in the future.

In the metastatic setting, the levels of cfDNA show a positive correlation with tumor burden [41]. There is evidence that detectable CTM in the blood among metastatic patients was an independent prognostic factor for shorter overall survival [58]. Gastric cancer-derived exosomal microRNAs and clonal mutations or CNVs detectable in ctDNA may contribute resistance to chemotherapy or HER2 inhibition, respectively [76,77,78,89,90,123]. Although there are limitations to CTC-based PD-L1 verification, there are other ways to determine PD-L1: from exosomal PD-L1 or to detect and quantify PD-L1 mRNA from ctRNA [104,105,107]. Data are conflicting and limited around cfDNA-based EBV detection and epigenomic applications [110,111]. If gastric cancer patients were also included in the phase II INSPIRE clinical trial, an analysis of the study may provide valuable information on tumor-based, personalized monitoring of the disease by ctDNA. Monitoring of cfDNA provided valuable information to differentiate between actual disease progression and radiological pseudo-progression, thus opening a window for immediate treatment changes in non-responders [113,116].

Most ongoing clinical trials address the pre- and post- surgical interval; two trials focus on the metastatic setting. The phase II GASTHER2 NCT04168931 trial examines the HER2 status of CTCs as a predictor of response to standard therapy combined with trastuzumab. The NCT04053725 trial investigates the predictive dynamics of serial ctDNA sampling during the immune-checkpoint blockade.

## 6. Conclusions

Recent revolutionary advancements in technology and the incorporation of genetic tumor characterization have significantly improved the possibilities of forecasting the prognosis of patients with metastatic gastric cancer, but the future holds even more excitement. Integrating broad tumor genomic characterization, dynamic monitoring of responses by the techniques of liquid biopsy will allow the implementation of adaptive, real-time treatment modifications in precision oncology. Clinical validation and standardization of novel liquid biopsy procedures are also necessary before being widely used in everyday practice. Although initial experiments analyzing liquid biopsies look very promising in patients with advanced gastric cancer, more prospective studies are needed to understand the molecular mechanisms behind resistance to targeted therapies. Applications of liquid biopsy to select and monitor patients receiving immunotherapy seem promising for identifying established and innovative qualitative–quantitative biomarkers in the ‘circulome’ and prompt treatment change in the primary- and secondary-resistant tumors. It seems that if the liquid biopsy and the immunotherapy revolution in cancer treatment eventually meet, they can facilitate patient compliance and improve overall outcomes.

## Figures and Tables

**Table 1:** Ongoing clinical trials using liquid biopsy approaches for gastric cancer.

Study Setting	Study Type (NCT Number and Trial Name)	Liquid Biopsy Approach	Estimated Enrollment	Primary Objectives	Estimated Primary Completion Date ^1^
Early stage	Prospective cohort (NCT04665687)	ctDNA	1730	To differentiate early gastric cancer and precancerous adenoma and predict recurrence by finding biomarkers through molecular profiling	December 2022
Neoadjuvant	Phase II (NCT03957564)	ctDNA, CTCs, and cfDNA	40	To evaluate CTC numbers/types, ctDNA mutation rate, cfDNA concentration and tumor response to neoadjuvant chemotherapy and surgery for resectable or locally advanced gastric or gastro-esophageal junction cancer patients	May 2024
Adjuvant	Phase II trial (NCT04510285)	ctDNA	48	To evaluate differences in 6-month ctDNA clearance rate in HER2+ esophagogastric cancer with persistent ctDNA following curative surgery when treated with “second adjuvant” trastuzumab with or without pembrolizumab	August 2022
Adjuvant and recurrence detection	Prospective cohort (NCT04000425)	ctDNA	55	To evaluate the role of ctDNA clearance during adjuvant chemotherapy (among patients with detectable ctDNA), and to define risk of recurrence in patients with newly detected positive ctDNA after radical gastrectomy	May 2021
Recurrence detection	Prospective cohort (NCT02887612)	ctDNA	200	To evaluate the positive predictive value of serum ctDNA positivity in the prediction of relapse after surgery in early and intermediate stage gastric cancer	June 2020
Advanced	Phase III trial (NCT03023436)	ctDNA and CTCs	220	To assess of ctDNA and CTC alterations as potential biomarkers for debulking surgery combined with hyperthermic intraperitoneal chemotherapy and systemic chemotherapy in patients with gastric cancer and peritoneal dissemination (as a secondary outcome measure)	June 2022
Advanced	Phase II (NCT04168931 GASTHER2)	CTCs	85	To investigate whether HER2-expressing CTCs may be suitable for prediction of response in patients with relapsed or metastatic gastric cancer who are histologically HER2-negative and treated with trastuzumab combination chemotherapy	January 2025
Advanced	Prospective cohort (NCT04053725)	ctDNA	200	To investigate the predictive dynamics of ctDNA mutation changes during immune-checkpoint blockade of gastric cancer patients	November 2021
All settings (from neoadjuvant to advanced)	Prospective cohort (NCT04576858 CURE)	ctDNA	1950	Prediction of prognosis and therapy response	July 2025

^1^ per Clinicaltrials.gov. (Accessed 24 March 2021).

## Data Availability

No new data were created or analyzed in this study. Data sharing is not applicable to this article.

## References

[1] Dasari A., Morris V.K., Allegra C.J., Atreya C., Benson A.B., Boland P., Chung K., Copur M.S., Corcoran R.B., Deming D.A. (2020). ctDNA applications and integration in colorectal cancer: An NCI Colon and Rectal-Anal Task Forces whitepaper. Nat. Rev. Clin. Oncol..

[2] Ignatiadis M., Sledge G.W., Jeffrey S.S. (2021). Liquid biopsy enters the clinic—Implementation issues and future challenges. Nat. Rev. Clin. Oncol..

[3] Rodríguez J., Avila J., Rolfo C., Ruíz-Patiño A., Russo A., Ricaurte L., Ordóñez-Reyes C., Arrieta O., Zatarain-Barrón Z.L., Recondo G. (2021). When Tissue is an Issue the Liquid Biopsy is Nonissue: A Review. Oncol. Ther..

[4] Bray F., Ferlay J., Soerjomataram I., Siegel R.L., Torre L.A., Jemal A. (2018). Global cancer statistics 2018: GLOBOCAN estimates of incidence and mortality worldwide for 36 cancers in 185 countries. CA Cancer J. Clin..

[5] World Health Organization Global Cancer Observatory. https://gco.iarc.fr/tomorrow/en/dataviz/isotype?cancers=7&single_unit=50000.

[6] Gobbi P.G., Bergonzi M., Pozzoli D., Villano L., Vanoli A., Corbella F., Dionigi P., Corazza G.R. (2011). Tumors of the gastroesophageal junction have intermediate prognosis compared to tumors of the esophagus and stomach, but share the same clinical determinants. Oncol. Lett..

[7] Cellini F., Morganti A.G., Di Matteo F.M., Mattiucci G.C., Valentini V. (2014). Clinical management of gastroesophageal junction tumors: Past and recent evidences for the role of radiotherapy in the multidisciplinary approach. Radiat. Oncol..

[8] Cancer Genome Atlas Research Network, Analysis Working Group: Asan University, BC Cancer Agency, Brigham and Women’s Hospital, Broad Institute, Brown University, Case Western Reserve University, Dana-Farber Cancer Institute, Duke University, Greater Poland Cancer Centre (2017). Integrated genomic characterization of oesophageal carcinoma. Nature.

[9] Kim J.W., Nam K.H., Ahn S.H., Park D.J., Kim H.H., Kim S.H., Chang H., Lee J.O., Kim Y.J., Lee H.S. (2016). Prognostic implications of immunosuppressive protein expression in tumors as well as immune cell infiltration within the tumor microenvironment in gastric cancer. Gastric Cancer.

[10] Zhang L., Qiu M., Jin Y., Ji J., Li B., Wang X., Yan S., Xu R., Yang D. (2015). Programmed cell death ligand 1 (PD-L1) expression on gastric cancer and its relationship with clinicopathologic factors. Int. J. Clin. Exp. Pathol..

[11] Bang Y.J., Van Cutsem E., Feyereislova A., Chung H.C., Shen L., Sawaki A., Lordick F., Ohtsu A., Omuro Y., Satoh T. (2010). Trastuzumab in combination with chemotherapy versus chemotherapy alone for treatment of HER2-positive advanced gastric or gastro-oesophageal junction cancer (ToGA): A phase 3, open-label, randomised controlled trial. Lancet.

[12] Boku N. (2014). HER2-positive gastric cancer. Gastric Cancer.

[13] Zhu L., Li Z., Wang Y., Zhang C., Liu Y., Qu X. (2015). Microsatellite instability and survival in gastric cancer: A systematic review and meta-analysis. Mol. Clin. Oncol..

[14] Smyth E.C., Wotherspoon A., Peckitt C., Gonzalez D., Hulkki-Wilson S., Eltahir Z., Fassan M., Rugge M., Valeri N., Okines A. (2017). Mismatch Repair Deficiency, Microsatellite Instability, and Survival: An Exploratory Analysis of the Medical Research Council Adjuvant Gastric Infusional Chemotherapy (MAGIC) Trial. JAMA Oncol..

[15] Kohlruss M., Grosser B., Krenauer M., Slotta-Huspenina J., Jesinghaus M., Blank S., Novotny A., Reiche M., Schmidt T., Ismani L. (2019). Prognostic implication of molecular subtypes and response to neoadjuvant chemotherapy in 760 gastric carcinomas: Role of Epstein-Barr virus infection and high- and low-microsatellite instability. J. Pathol. Clin. Res..

[16] Polom K., Marano L., Marrelli D., De Luca R., Roviello G., Savelli V., Tan P., Roviello F. (2018). Meta-analysis of microsatellite instability in relation to clinicopathological characteristics and overall survival in gastric cancer. Br. J. Surg..

[17] Puliga E., Corso S., Pietrantonio F., Giordano S. (2021). Microsatellite instability in Gastric Cancer: Between lights and shadows. Cancer Treat. Rev..

[18] Ma C., Patel K., Singhi A.D., Ren B., Zhu B., Shaikh F., Sun W. (2016). Programmed Death-Ligand 1 Expression Is Common in Gastric Cancer Associated With Epstein-Barr Virus or Microsatellite Instability. Am. J. Surg. Pathol..

[19] Derks S., Liao X., Chiaravalli A.M., Xu X., Camargo M.C., Solcia E., Sessa F., Fleitas T., Freeman G.J., Rodig S.J. (2016). Abundant PD-L1 expression in Epstein-Barr Virus-infected gastric cancers. Oncotarget.

[20] Fuchs C.S., Doi T., Jang R.W., Muro K., Satoh T., Machado M., Sun W., Jalal S.I., Shah M.A., Metges J.P. (2018). Safety and Efficacy of Pembrolizumab Monotherapy in Patients With Previously Treated Advanced Gastric and Gastroesophageal Junction Cancer: Phase 2 Clinical KEYNOTE-059 Trial. JAMA Oncol..

[21] Shitara K., Özgüroğlu M., Bang Y.J., Di Bartolomeo M., Mandalà M., Ryu M.H., Fornaro L., Olesiński T., Caglevic C., Chung H.C. (2018). Pembrolizumab versus paclitaxel for previously treated, advanced gastric or gastro-oesophageal junction cancer (KEYNOTE-061): A randomised, open-label, controlled, phase 3 trial. Lancet.

[22] Tabernero J., Cutsem E.V., Bang Y.-J., Fuchs C.S., Wyrwicz L., Lee K.W., Kudaba I., Garrido M., Chung H.C., Salguero H.R.C. (2019). Pembrolizumab with or without chemotherapy versus chemotherapy for advanced gastric or gastroesophageal junction (G/GEJ) adenocarcinoma: The phase III KEYNOTE-062 study. J. Clin. Oncol..

[23] National Comprehensive Cancer Network Gastric Cancer (Version 1.2021). https://www.nccn.org/professionals/physician_gls/default.aspx#gastric.

[24] Shitara K., Özgüroğlu M., Bang Y.-J., Bartolomeo M.D., Mandalà M., Ryu M.-h., Vivaldi C., Olesinski T., Chung H.C., Muro K. (2020). The association of tissue tumor mutational burden (tTMB) using the Foundation Medicine genomic platform with efficacy of pembrolizumab versus paclitaxel in patients (pts) with gastric cancer (GC) from KEYNOTE-061. J. Clin. Oncol..

[25] Fuchs C.S., Özgüroğlu M., Bang Y.-J., Bartolomeo M.D., Mandalà M., Ryu M.-h., Fornaro L., Olesinski T., Caglevic C., Chung H.C. (2020). Pembrolizumab versus paclitaxel for previously treated patients with PD-L1–positive advanced gastric or gastroesophageal junction cancer (GC): Update from the phase III KEYNOTE-061 trial. J. Clin. Oncol..

[26] Kim S.T., Cristescu R., Bass A.J., Kim K.M., Odegaard J.I., Kim K., Liu X.Q., Sher X., Jung H., Lee M. (2018). Comprehensive molecular characterization of clinical responses to PD-1 inhibition in metastatic gastric cancer. Nat. Med..

[27] Mandel P., Metais P. (1948). [Nuclear Acids In Human Blood Plasma]. Comptes Rendus Seances Soc. Biol. Fil..

[28] Leon S.A., Shapiro B., Sklaroff D.M., Yaros M.J. (1977). Free DNA in the serum of cancer patients and the effect of therapy. Cancer Res..

[29] Sorenson G.D., Pribish D.M., Valone F.H., Memoli V.A., Bzik D.J., Yao S.L. (1994). Soluble normal and mutated DNA sequences from single-copy genes in human blood. Cancer Epidemiol. Biomark. Prev..

[30] Vasioukhin V., Anker P., Maurice P., Lyautey J., Lederrey C., Stroun M. (1994). Point mutations of the N-ras gene in the blood plasma DNA of patients with myelodysplastic syndrome or acute myelogenous leukaemia. Br. J. Haematol..

[31] Chen X.Q., Stroun M., Magnenat J.L., Nicod L.P., Kurt A.M., Lyautey J., Lederrey C., Anker P. (1996). Microsatellite alterations in plasma DNA of small cell lung cancer patients. Nat. Med..

[32] Diehl F., Schmidt K., Choti M.A., Romans K., Goodman S., Li M., Thornton K., Agrawal N., Sokoll L., Szabo S.A. (2008). Circulating mutant DNA to assess tumor dynamics. Nat. Med..

[33] Wang Y., Springer S., Mulvey C.L., Silliman N., Schaefer J., Sausen M., James N., Rettig E.M., Guo T., Pickering C.R. (2015). Detection of somatic mutations and HPV in the saliva and plasma of patients with head and neck squamous cell carcinomas. Sci. Transl. Med..

[34] Kimura H., Fujiwara Y., Sone T., Kunitoh H., Tamura T., Kasahara K., Nishio K. (2006). EGFR mutation status in tumour-derived DNA from pleural effusion fluid is a practical basis for predicting the response to gefitinib. Br. J. Cancer.

[35] Diehl F., Schmidt K., Durkee K.H., Moore K.J., Goodman S.N., Shuber A.P., Kinzler K.W., Vogelstein B. (2008). Analysis of mutations in DNA isolated from plasma and stool of colorectal cancer patients. Gastroenterology.

[36] De Mattos-Arruda L., Mayor R., Ng C.K.Y., Weigelt B., Martínez-Ricarte F., Torrejon D., Oliveira M., Arias A., Raventos C., Tang J. (2015). Cerebrospinal fluid-derived circulating tumour DNA better represents the genomic alterations of brain tumours than plasma. Nat. Commun..

[37] Poulet G., Massias J., Taly V. (2019). Liquid Biopsy: General Concepts. Acta Cytol..

[38] In ‘t Veld S.G.J.G., Wurdinger T. (2019). Tumor-educated platelets. Blood.

[39] Heitzer E., Auinger L., Speicher M.R. (2020). Cell-Free DNA and Apoptosis: How Dead Cells Inform About the Living. Trends Mol. Med..

[40] Schwarzenbach H., Hoon D.S., Pantel K. (2011). Cell-free nucleic acids as biomarkers in cancer patients. Nat. Rev. Cancer.

[41] Kim K., Shin D.G., Park M.K., Baik S.H., Kim T.H., Kim S., Lee S. (2014). Circulating cell-free DNA as a promising biomarker in patients with gastric cancer: Diagnostic validity and significant reduction of cfDNA after surgical resection. Ann. Surg. Treat. Res..

[42] Lan Y.T., Chen M.H., Fang W.L., Hsieh C.C., Lin C.H., Jhang F.Y., Yang S.H., Lin J.K., Chen W.S., Jiang J.K. (2017). Clinical relevance of cell-free DNA in gastrointestinal tract malignancy. Oncotarget.

[43] Pu W.Y., Zhang R., Xiao L., Wu Y.Y., Gong W., Lv X.D., Zhong F.Y., Zhuang Z.X., Bai X.M., Li K. (2016). Prediction of cancer progression in a group of 73 gastric cancer patients by circulating cell-free DNA. BMC Cancer.

[44] Olsson E., Winter C., George A., Chen Y., Howlin J., Tang M.H., Dahlgren M., Schulz R., Grabau D., van Westen D. (2015). Serial monitoring of circulating tumor DNA in patients with primary breast cancer for detection of occult metastatic disease. EMBO Mol. Med..

[45] Reinert T., Schøler L.V., Thomsen R., Tobiasen H., Vang S., Nordentoft I., Lamy P., Kannerup A.S., Mortensen F.V., Stribolt K. (2016). Analysis of circulating tumour DNA to monitor disease burden following colorectal cancer surgery. Gut.

[46] Tie J., Cohen J.D., Wang Y., Christie M., Simons K., Lee M., Wong R., Kosmider S., Ananda S., McKendrick J. (2019). Circulating Tumor DNA Analyses as Markers of Recurrence Risk and Benefit of Adjuvant Therapy for Stage III Colon Cancer. JAMA Oncol..

[47] Hamakawa T., Kukita Y., Kurokawa Y., Miyazaki Y., Takahashi T., Yamasaki M., Miyata H., Nakajima K., Taniguchi K., Takiguchi S. (2015). Monitoring gastric cancer progression with circulating tumour DNA. Br. J. Cancer.

[48] Yang J., Gong Y., Lam V.K., Shi Y., Guan Y., Zhang Y., Ji L., Chen Y., Zhao Y., Qian F. (2020). Deep sequencing of circulating tumor DNA detects molecular residual disease and predicts recurrence in gastric cancer. Cell Death Dis..

[49] Kim Y.-W., Kim Y.-H., Song Y., Kim H.-S., Sim H.W., Poojan S., Eom B.W., Kook M.-C., Joo J., Hong K.-M. (2019). Monitoring circulating tumor DNA by analyzing personalized cancer-specific rearrangements to detect recurrence in gastric cancer. Exp. Mol. Med..

[50] Nakamura Y., Fujisawa T., Kadowaki S., Takahashi N., Goto M., Yoshida K., Kawakami T., Esaki T., Oki E., Nishida N. (2021). Characteristics of genomic alterations in circulating tumor DNA (ctDNA) in patients (Pts) with advanced gastrointestinal (GI) cancers in nationwide large-scale ctDNA screening:SCRUM-Japan Monstar-Screen. J. Clin. Oncol..

[51] Nakamura Y., Taniguchi H., Ikeda M., Bando H., Kato K., Morizane C., Esaki T., Komatsu Y., Kawamoto Y., Takahashi N. (2020). Clinical utility of circulating tumor DNA sequencing in advanced gastrointestinal cancer: SCRUM-Japan GI-SCREEN and GOZILA studies. Nat. Med..

[52] Pantel K., Alix-Panabières C. (2017). Liquid biopsy in 2016: Circulating tumour cells and cell-free DNA in gastrointestinal cancer. Nat. Rev. Gastroenterol. Hepatol..

[53] Huang X., Gao P., Sun J., Chen X., Song Y., Zhao J., Xu H., Wang Z. (2015). Clinicopathological and prognostic significance of circulating tumor cells in patients with gastric cancer: A meta-analysis. Int. J. Cancer.

[54] Zhang Q., Shan F., Li Z., Gao J., Li Y., Shen L., Ji J., Lu M. (2018). A prospective study on the changes and clinical significance of pre-operative and post-operative circulating tumor cells in resectable gastric cancer. J. Transl. Med..

[55] Szczepanik A., Sierzega M., Drabik G., Pituch-Noworolska A., Kołodziejczyk P., Zembala M. (2019). CD44(+) cytokeratin-positive tumor cells in blood and bone marrow are associated with poor prognosis of patients with gastric cancer. Gastric Cancer.

[56] Li Y., Zhang X., Ge S., Gao J., Gong J., Lu M., Zhang Q., Cao Y., Wang D.D., Lin P.P. (2014). Clinical significance of phenotyping and karyotyping of circulating tumor cells in patients with advanced gastric cancer. Oncotarget.

[57] Li Y., Gong J., Zhang Q., Lu Z., Gao J., Li Y., Cao Y., Shen L. (2016). Dynamic monitoring of circulating tumour cells to evaluate therapeutic efficacy in advanced gastric cancer. Br. J. Cancer.

[58] Zheng X., Fan L., Zhou P., Ma H., Huang S., Yu D., Zhao L., Yang S., Liu J., Huang A. (2017). Detection of Circulating Tumor Cells and Circulating Tumor Microemboli in Gastric Cancer. Transl. Oncol..

[59] Gorges T.M., Tinhofer I., Drosch M., Röse L., Zollner T.M., Krahn T., von Ahsen O. (2012). Circulating tumour cells escape from EpCAM-based detection due to epithelial-to-mesenchymal transition. BMC Cancer.

[60] Gazzaniga P., Naso G., Gradilone A., Cortesi E., Gandini O., Gianni W., Fabbri M.A., Vincenzi B., di Silverio F., Frati L. (2010). Chemosensitivity profile assay of circulating cancer cells: Prognostic and predictive value in epithelial tumors. Int. J. Cancer.

[61] Wang Z., Chen J.-q., Liu J.-l., Tian L. (2019). Issues on peritoneal metastasis of gastric cancer: An update. World J. Surg. Oncol..

[62] Deng G., Qu J., Zhang Y., Che X., Cheng Y., Fan Y., Zhang S., Na D., Liu Y., Qu X. (2017). Gastric cancer-derived exosomes promote peritoneal metastasis by destroying the mesothelial barrier. FEBS Lett..

[63] Keerthikumar S., Chisanga D., Ariyaratne D., Al Saffar H., Anand S., Zhao K., Samuel M., Pathan M., Jois M., Chilamkurti N. (2016). ExoCarta: A Web-Based Compendium of Exosomal Cargo. J. Mol. Biol..

[64] Qu J.L., Qu X.J., Zhao M.F., Teng Y.E., Zhang Y., Hou K.Z., Jiang Y.H., Yang X.H., Liu Y.P. (2009). Gastric cancer exosomes promote tumour cell proliferation through PI3K/Akt and MAPK/ERK activation. Dig. Liver Dis..

[65] Li C., Liu D.-R., Li G.-G., Wang H.-H., Li X.-W., Zhang W., Wu Y.-L., Chen L. (2015). CD97 promotes gastric cancer cell proliferation and invasion through exosome-mediated MAPK signaling pathway. World J. Gastroenterol..

[66] Zhang Y., Han T., Feng D., Li J., Wu M., Peng X., Wang B., Zhan X., Fu P. (2020). Screening of non-invasive miRNA biomarker candidates for metastasis of gastric cancer by small RNA sequencing of plasma exosomes. Carcinogenesis.

[67] Zhu M., Zhang N., He S., Lu X. (2020). Exosomal miR-106a derived from gastric cancer promotes peritoneal metastasis via direct regulation of Smad7. Cell Cycle.

[68] Ohzawa H., Saito A., Kumagai Y., Kimura Y., Yamaguchi H., Hosoya Y., Lefor A.K., Sata N., Kitayama J. (2020). Reduced expression of exosomal miR-29s in peritoneal fluid is a useful predictor of peritoneal recurrence after curative resection of gastric cancer with serosal involvement. Oncol. Rep..

[69] Kersy O., Loewenstein S., Lubezky N., Sher O., Simon N.B., Klausner J.M., Lahat G. (2019). Omental Tissue-Mediated Tumorigenesis of Gastric Cancer Peritoneal Metastases. Front. Oncol..

[70] Sohel M.M.H. (2020). Circulating microRNAs as biomarkers in cancer diagnosis. Life Sci..

[71] Fuji T., Umeda Y., Nyuya A., Taniguchi F., Kawai T., Yasui K., Toshima T., Yoshida K., Fujiwara T., Goel A. (2019). Detection of circulating microRNAs with Ago2 complexes to monitor the tumor dynamics of colorectal cancer patients during chemotherapy. Int. J. Cancer.

[72] Zhang J., Fan X.S., Wang C.X., Liu B., Li Q., Zhou X.J. (2013). Up-regulation of Ago2 expression in gastric carcinoma. Med. Oncol..

[73] Umer M., Vaidyanathan R., Nguyen N.T., Shiddiky M.J.A. (2018). Circulating tumor microemboli: Progress in molecular understanding and enrichment technologies. Biotechnol. Adv..

[74] Hou J.M., Krebs M., Ward T., Sloane R., Priest L., Hughes A., Clack G., Ranson M., Blackhall F., Dive C. (2011). Circulating tumor cells as a window on metastasis biology in lung cancer. Am. J. Pathol..

[75] Ding X.-Q., Wang Z.-Y., Xia D., Wang R.-X., Pan X.-R., Tong J.-H. (2020). Proteomic Profiling of Serum Exosomes From Patients With Metastatic Gastric Cancer. Front. Oncol..

[76] Sun M.Y., Xu B., Wu Q.X., Chen W.L., Cai S., Zhang H., Tang Q.F. (2021). Cisplatin-Resistant Gastric Cancer Cells Promote the Chemoresistance of Cisplatin-Sensitive Cells via the Exosomal RPS3-Mediated PI3K-Akt-Cofilin-1 Signaling Axis. Front. Cell Dev. Biol..

[77] Xu Y.C., Liu X., Li M., Li Y., Li C.Y., Lu Y., Sanches J., Wang L., Du Y., Mao L.M. (2018). A Novel Mechanism of Doxorubicin Resistance and Tumorigenesis Mediated by MicroRNA-501-5p-Suppressed BLID. Mol. Ther. Nucleic Acids.

[78] Zhao W., Shan B., He D., Cheng Y., Li B., Zhang C., Duan C. (2019). Recent Progress in Characterizing Long Noncoding RNAs in Cancer Drug Resistance. J. Cancer.

[79] Fuchs C.S., Tabernero J., Tomášek J., Chau I., Melichar B., Safran H., Tehfe M.A., Filip D., Topuzov E., Schlittler L. (2016). Biomarker analyses in REGARD gastric/GEJ carcinoma patients treated with VEGFR2-targeted antibody ramucirumab. Br. J. Cancer.

[80] Van Cutsem E., de Haas S., Kang Y.K., Ohtsu A., Tebbutt N.C., Ming Xu J., Peng Yong W., Langer B., Delmar P., Scherer S.J. (2012). Bevacizumab in combination with chemotherapy as first-line therapy in advanced gastric cancer: A biomarker evaluation from the AVAGAST randomized phase III trial. J. Clin. Oncol..

[81] Zhao D., Hou H., Zhang X. (2018). Progress in the treatment of solid tumors with apatinib: A systematic review. OncoTargets Ther..

[82] Abdallah E.A., Braun A.C., Flores B., Senda L., Urvanegia A.C., Calsavara V., Fonseca de Jesus V.H., Almeida M.F.A., Begnami M.D., Coimbra F.J.F. (2019). The Potential Clinical Implications of Circulating Tumor Cells and Circulating Tumor Microemboli in Gastric Cancer. Oncologist.

[83] Mitani S., Kawakami H. (2020). Emerging Targeted Therapies for HER2 Positive Gastric Cancer That Can Overcome Trastuzumab Resistance. Cancers.

[84] Marin J.J.G., Perez-Silva L., Macias R.I.R., Asensio M., Peleteiro-Vigil A., Sanchez-Martin A., Cives-Losada C., Sanchon-Sanchez P., Sanchez De Blas B., Herraez E. (2020). Molecular Bases of Mechanisms Accounting for Drug Resistance in Gastric Adenocarcinoma. Cancers.

[85] Piro G., Carbone C., Cataldo I., Di Nicolantonio F., Giacopuzzi S., Aprile G., Simionato F., Boschi F., Zanotto M., Mina M.M. (2016). An FGFR3 Autocrine Loop Sustains Acquired Resistance to Trastuzumab in Gastric Cancer Patients. Clin. Cancer Res..

[86] Shi J., Li F., Yao X., Mou T., Xu Z., Han Z., Chen S., Li W., Yu J., Qi X. (2018). The HER4-YAP1 axis promotes trastuzumab resistance in HER2-positive gastric cancer by inducing epithelial and mesenchymal transition. Oncogene.

[87] Sampera A., Sánchez-Martín F.J., Arpí O., Visa L., Iglesias M., Menéndez S., Gaye É., Dalmases A., Clavé S., Gelabert-Baldrich M. (2019). HER-Family Ligands Promote Acquired Resistance to Trastuzumab in Gastric Cancer. Mol. Cancer Ther..

[88] Liu J., Pan C., Guo L., Wu M., Guo J., Peng S., Wu Q., Zuo Q. (2016). A new mechanism of trastuzumab resistance in gastric cancer: MACC1 promotes the Warburg effect via activation of the PI3K/AKT signaling pathway. J. Hematol. Oncol..

[89] Wang Y., Zhao C., Chang L., Jia R., Liu R., Zhang Y., Gao X., Li J., Chen R., Xia X. (2019). Circulating tumor DNA analyses predict progressive disease and indicate trastuzumab-resistant mechanism in advanced gastric cancer. EBioMedicine.

[90] Wang D.-S., Liu Z.-X., Lu Y.-X., Bao H., Wu X., Zeng Z.-L., Liu Z., Zhao Q., He C.-Y., Lu J.-H. (2019). Liquid biopsies to track trastuzumab resistance in metastatic HER2-positive gastric cancer. Gut.

[91] Shitara K., Van Cutsem E., Bang Y.J., Fuchs C., Wyrwicz L., Lee K.W., Kudaba I., Garrido M., Chung H.C., Lee J. (2020). Efficacy and Safety of Pembrolizumab or Pembrolizumab Plus Chemotherapy vs Chemotherapy Alone for Patients With First-line, Advanced Gastric Cancer: The KEYNOTE-062 Phase 3 Randomized Clinical Trial. JAMA Oncol..

[92] Moehler M., Shitara K., Garrido M., Salman P., Shen L., Wyrwicz L., Yamaguchi K., Skoczylas T., Campos Bragagnoli A., Liu T. (2020). LBA6_PR Nivolumab (nivo) plus chemotherapy (chemo) versus chemo as first-line (1L) treatment for advanced gastric cancer/gastroesophageal junction cancer (GC/GEJC)/esophageal adenocarcinoma (EAC): First results of the CheckMate 649 study. Ann. Oncol..

[93] Sundar R., Smyth E.C., Peng S., Yeong J.P.S., Tan P. (2020). Predictive Biomarkers of Immune Checkpoint Inhibition in Gastroesophageal Cancers. Front. Oncol..

[94] Folprecht G. (2019). Tumor mutational burden as a new biomarker for PD-1 antibody treatment in gastric cancer. Cancer Commun..

[95] Snyder A., Morrissey M.P., Hellmann M.D. (2019). Use of Circulating Tumor DNA for Cancer Immunotherapy. Clin. Cancer Res..

[96] Marabelle A., Fakih M., Lopez J., Shah M., Shapira-Frommer R., Nakagawa K., Chung H.C., Kindler H.L., Lopez-Martin J.A., Miller W.H. (2020). Association of tumour mutational burden with outcomes in patients with advanced solid tumours treated with pembrolizumab: Prospective biomarker analysis of the multicohort, open-label, phase 2 KEYNOTE-158 study. Lancet Oncol..

[97] Wang F., Wei X.L., Wang F.H., Xu N., Shen L., Dai G.H., Yuan X.L., Chen Y., Yang S.J., Shi J.H. (2019). Safety, efficacy and tumor mutational burden as a biomarker of overall survival benefit in chemo-refractory gastric cancer treated with toripalimab, a PD-1 antibody in phase Ib/II clinical trial NCT02915432. Ann. Oncol..

[98] Cho J., Ahn S., Son D.S., Kim N.K., Lee K.W., Kim S., Lee J., Park S.H., Park J.O., Kang W.K. (2019). Bridging genomics and phenomics of gastric carcinoma. Int. J. Cancer.

[99] Gandara D.R., Paul S.M., Kowanetz M., Schleifman E., Zou W., Li Y., Rittmeyer A., Fehrenbacher L., Otto G., Malboeuf C. (2018). Blood-based tumor mutational burden as a predictor of clinical benefit in non-small-cell lung cancer patients treated with atezolizumab. Nat. Med..

[100] Kim J., Kim B., Kang S.Y., Heo Y.J., Park S.H., Kim S.T., Kang W.K., Lee J., Kim K.M. (2020). Tumor Mutational Burden Determined by Panel Sequencing Predicts Survival After Immunotherapy in Patients With Advanced Gastric Cancer. Front. Oncol..

[101] Sohn B.H., Hwang J.E., Jang H.J., Lee H.S., Oh S.C., Shim J.J., Lee K.W., Kim E.H., Yim S.Y., Lee S.H. (2017). Clinical Significance of Four Molecular Subtypes of Gastric Cancer Identified by The Cancer Genome Atlas Project. Clin. Cancer Res..

[102] Morihiro T., Kuroda S., Kanaya N., Kakiuchi Y., Kubota T., Aoyama K., Tanaka T., Kikuchi S., Nagasaka T., Nishizaki M. (2019). PD-L1 expression combined with microsatellite instability/CD8+ tumor infiltrating lymphocytes as a useful prognostic biomarker in gastric cancer. Sci. Rep..

[103] Pietrantonio F., Miceli R., Raimondi A., Kim Y.W., Kang W.K., Langley R.E., Choi Y.Y., Kim K.M., Nankivell M.G., Morano F. (2019). Individual Patient Data Meta-Analysis of the Value of Microsatellite Instability As a Biomarker in Gastric Cancer. J. Clin. Oncol..

[104] Schehr J.L., Schultz Z.D., Warrick J.W., Guckenberger D.J., Pezzi H.M., Sperger J.M., Heninger E., Saeed A., Leal T., Mattox K. (2016). High Specificity in Circulating Tumor Cell Identification Is Required for Accurate Evaluation of Programmed Death-Ligand 1. PLoS ONE.

[105] Fan Y., Che X., Qu J., Hou K., Wen T., Li Z., Li C., Wang S., Xu L., Liu Y. (2019). Exosomal PD-L1 Retains Immunosuppressive Activity and is Associated with Gastric Cancer Prognosis. Ann. Surg. Oncol..

[106] Ayala-Mar S., Donoso-Quezada J., González-Valdez J. (2021). Clinical Implications of Exosomal PD-L1 in Cancer Immunotherapy. J. Immunol. Res..

[107] Ishiba T., Hoffmann A.C., Usher J., Elshimali Y., Sturdevant T., Dang M., Jaimes Y., Tyagi R., Gonzales R., Grino M. (2018). Frequencies and expression levels of programmed death ligand 1 (PD-L1) in circulating tumor RNA (ctRNA) in various cancer types. Biochem. Biophys. Res. Commun..

[108] Mayrhofer M., De Laere B., Whitington T., Van Oyen P., Ghysel C., Ampe J., Ost P., Demey W., Hoekx L., Schrijvers D. (2018). Cell-free DNA profiling of metastatic prostate cancer reveals microsatellite instability, structural rearrangements and clonal hematopoiesis. Genome Med..

[109] Willis J., Lefterova M.I., Artyomenko A., Kasi P.M., Nakamura Y., Mody K., Catenacci D.V.T., Fakih M., Barbacioru C., Zhao J. (2019). Validation of Microsatellite Instability Detection Using a Comprehensive Plasma-Based Genotyping Panel. Clin. Cancer Res..

[110] Shoda K., Ichikawa D., Fujita Y., Masuda K., Hiramoto H., Hamada J., Arita T., Konishi H., Kosuga T., Komatsu S. (2017). Clinical utility of circulating cell-free Epstein-Barr virus DNA in patients with gastric cancer. Oncotarget.

[111] Ignatova E., Seriak D., Fedyanin M., Tryakin A., Pokataev I., Menshikova S., Vakhabova Y., Smirnova K., Tjulandin S., Ajani J.A. (2020). Epstein-Barr virus-associated gastric cancer: Disease that requires special approach. Gastric Cancer.

[112] Cao Y., Xie L., Shi F., Tang M., Li Y., Hu J., Zhao L., Zhao L., Yu X., Luo X. (2021). Targeting the signaling in Epstein-Barr virus-associated diseases: Mechanism, regulation, and clinical study. Signal Transduct. Target. Ther..

[113] Bratman S.V., Yang S.Y.C., Iafolla M.A.J., Liu Z., Hansen A.R., Bedard P.L., Lheureux S., Spreafico A., Razak A.A., Shchegrova S. (2020). Personalized circulating tumor DNA analysis as a predictive biomarker in solid tumor patients treated with pembrolizumab. Nat. Cancer.

[114] Matsusaka S., Chìn K., Ogura M., Suenaga M., Shinozaki E., Mishima Y., Terui Y., Mizunuma N., Hatake K. (2010). Circulating tumor cells as a surrogate marker for determining response to chemotherapy in patients with advanced gastric cancer. Cancer Sci..

[115] Uenosono Y., Arigami T., Kozono T., Yanagita S., Hagihara T., Haraguchi N., Matsushita D., Hirata M., Arima H., Funasako Y. (2013). Clinical significance of circulating tumor cells in peripheral blood from patients with gastric cancer. Cancer.

[116] Jensen T.J., Goodman A.M., Kato S., Ellison C.K., Daniels G.A., Kim L., Nakashe P., McCarthy E., Mazloom A.R., McLennan G. (2019). Genome-Wide Sequencing of Cell-Free DNA Identifies Copy-Number Alterations That Can Be Used for Monitoring Response to Immunotherapy in Cancer Patients. Mol. Cancer Ther..

[117] Ye M., Huang D., Zhang Q., Weng W., Tan C., Qin G., Jiang W., Sheng W., Wang L. (2020). Heterogeneous programmed death-ligand 1 expression in gastric cancer: Comparison of tissue microarrays and whole sections. Cancer Cell Int..

[118] Vogelstein B., Papadopoulos N., Velculescu V.E., Zhou S., Diaz L.A., Kinzler K.W. (2013). Cancer Genome Landscapes. Science.

[119] Uchôa Guimarães C.T., Ferreira Martins N.N., Cristina da Silva Oliveira K., Almeida C.M., Pinheiro T.M., Gigek C.O., Roberto de Araújo Cavallero S., Assumpção P.P., Cardoso Smith M.A., Burbano R.R. (2018). Liquid biopsy provides new insights into gastric cancer. Oncotarget.

[120] Crowley E., Di Nicolantonio F., Loupakis F., Bardelli A. (2013). Liquid biopsy: Monitoring cancer-genetics in the blood. Nat. Rev. Clin. Oncol..

[121] Necula L., Matei L., Dragu D., Neagu A.I., Mambet C., Nedeianu S., Bleotu C., Diaconu C.C., Chivu-Economescu M. (2019). Recent advances in gastric cancer early diagnosis. World J. Gastroenterol..

[122] Yang C., Chen F., Wang S., Xiong B. (2019). Circulating Tumor Cells in Gastrointestinal Cancers: Current Status and Future Perspectives. Front. Oncol..

[123] Liu X., Lu Y., Xu Y., Hou S., Huang J., Wang B., Zhao J., Xia S., Fan S., Yu X. (2019). Exosomal transfer of miR-501 confers doxorubicin resistance and tumorigenesis via targeting of BLID in gastric cancer. Cancer Lett..

